# Community-Based Preventive Health Interventions and Their Impact on Population Health Outcomes: A Narrative Review

**DOI:** 10.7759/cureus.108091

**Published:** 2026-05-01

**Authors:** Prashant Vinayakrao Solanke, PM Shilpa, Komal Pukur Thekdi, PGN Swamy, Kirti M Jamdar, Priti Sriranjan

**Affiliations:** 1 Department of Community Medicine, Dr Ulhas Patil Medical College, Jalgaon, IND; 2 Department of Pediatric Nursing, Adichunchanagiri College of Nursing, Adichunchanagiri University, Balagangadharanatha Nagara, IND; 3 Department of Community Medicine, Dr ND Desai Faculty of Medical Sciences and Research, Dharmsinh Desai University, Nadiad, IND; 4 Department of Community Health Nursing, Adichunchanagiri College of Nursing, Adichunchanagiri University, Balagangadharanatha Nagara, IND; 5 Department of Nursing, Shreemati Nathibai Damodar Thackersey Women's University, Mumbai, IND; 6 Department of Psychology, Narasingh Choudhury Autonomous College, Jajpur, IND

**Keywords:** behavioral modification, community engagement, health equity, population health, preventive interventions

## Abstract

Community-based preventive health interventions have emerged as central strategies for improving population health and reducing preventable morbidity and mortality across diverse communities. These approaches extend beyond clinical care and incorporate behavioral, environmental, social, and structural components delivered within local settings. Despite wide implementation, knowledge remains dispersed across intervention types, with limited integration of long-term outcomes and equity-focused perspectives. This narrative review synthesizes current understanding of major categories of community-based preventive strategies, including health promotion, screening and early detection, community engagement mechanisms, workforce models such as community health workers, digital and mobile health solutions including social media-based interventions, and policy and environmental actions. A structured narrative approach was used to examine conceptual foundations, implementation characteristics, and reported impacts across varied geographic and socioeconomic contexts. Integrated, multicomponent initiatives that combine behavioral modification, participatory engagement, decentralized screening, and supportive structural measures are associated with improvements in risk factor management, service accessibility, and early identification of chronic conditions. Variations in contextual adaptation, governance arrangements, financing stability, and workforce capacity influence the magnitude and sustainability of outcomes. Stronger coordination across sectors, standardized monitoring indicators, and closer alignment with primary healthcare systems are necessary to enhance scalability and long-term impact. Community-based prevention provides a viable pathway for advancing equitable improvements in population health when designed and locally responsive.

## Introduction and background

Preventive health interventions encompassing local, community-driven activities have become key elements of modern public health policies that may enhance health outcomes and reduce preventable morbidity and mortality across diverse populations [1]. These strategies are not limited to clinical care but extend to behavioral, environmental, social, and structural determinants operating at individual, interpersonal, organizational, and societal levels [2]. The shift from treatment-based systems to prevention-oriented models reflects increasing recognition that social factors such as income, education, housing quality, food access, employment status, and neighborhood infrastructure strongly influence disease prevalence and life expectancy [3]. Population health frameworks therefore emphasize upstream actions and collective responsibility to address modifiable risk factors linked to non-communicable diseases such as cardiovascular disease, diabetes, chronic respiratory diseases, and cancer, as well as communicable diseases prevalent in low- and middle-income regions and underserved populations [4].

These interventions are grounded in local realities and are developed through participatory planning processes aligned with local priorities, enhancing contextual relevance and social acceptability [5]. Collaboration among public health institutions, local organizations, civil society groups, and leadership structures strengthens coordination and supports culturally appropriate program delivery [6]. Such involvement fosters trust, collective ownership, and accountability, thereby improving participation and long-term maintenance of preventive efforts [7]. This model emphasizes bottom-up engagement rather than top-down implementation. Frontline health workers and lay facilitators play a key role in bridging gaps between formal healthcare systems and underserved populations by providing outreach services, culturally competent education, risk assessment, screening, navigation, and referral support [8]. Their proximity to populations enhances communication, reduces perceived barriers, and improves access to preventive services. Health promotion activities implemented in schools, workplaces, faith-based organizations, and neighborhood centers contribute to improvements in physical activity, dietary practices, smoking cessation, weight management, and reduction of harmful alcohol use across different population groups [9]. Embedding preventive initiatives within existing local structures aligns with broader goals of universal health coverage and equitable access to essential services, particularly in resource-constrained settings where structural barriers persist [10].

Adaptation, monitoring, and scaling of preventive strategies across diverse sociocultural and economic contexts are guided by implementation frameworks [11,12]. Conceptual models such as the socio-ecological framework and RE-AIM (reach, effectiveness, adoption, implementation, and maintenance) explain how individual behavior interacts with interpersonal networks, institutional environments, and policy conditions to influence program reach and sustainability [13]. These interrelated mechanisms support the development of integrated interventions that address behavioral, environmental, and structural determinants simultaneously. Such approaches contribute to reductions in cardiovascular risk factors, improvements in glycemic control, increased vaccination coverage, enhanced maternal and child health outcomes, and earlier detection of chronic conditions through coordinated delivery systems [14]. Local programs also facilitate detection of undiagnosed hypertension, diabetes, and certain cancers, ensuring timely linkage to care and follow-up [15]. Exposure to risk-promoting environments is reduced through regulatory and environmental measures, including fiscal strategies and modifications to the built environment [16]. Digital platforms and mobile health technologies, including social media-based tools, further enhance outreach, health literacy, remote monitoring, and coordination between providers and participants [17].

Variations in contextual adaptation, workforce capacity, leadership engagement, financing structures, and governance systems influence the consistency and equity of outcomes across settings [18]. Multisectoral collaboration, institutional commitment, integration with primary healthcare systems, and continuous capacity building are essential for achieving sustainable population-level gains [6]. Long-term impact depends on sustained implementation, local ownership, and equitable resource distribution. The existing evidence base remains fragmented across intervention types and geographic contexts, limiting direct comparison of long-term outcomes. Inconsistencies in standardized indicators and limited emphasis on equity further constrain synthesis. Greater integration of behavioral, environmental, and structural evidence is required to support scalable and context-sensitive prevention models.

Objective of the review

This narrative review aims to summarize existing evidence on community-based preventive interventions and analyze their effects on population health outcomes across diverse settings, including low- and middle-income countries, high-income contexts, and vulnerable populations. The review examines intervention types, implementation strategies, and reported health indicators, with emphasis on identifying cross-context patterns and equity considerations. It also seeks to identify gaps in the literature and evaluate factors influencing effectiveness, scalability, and sustainability, while providing an integrated synthesis that extends beyond prior studies focused on single intervention categories or specific disease areas.

Methodology

A structured narrative review methodology was adopted to ensure transparency and reproducibility. This study is explicitly designed as a narrative review and does not follow the full methodological framework of a systematic review. A comprehensive literature search was conducted across major electronic databases, including PubMed, Scopus, and Web of Science, supplemented by manual searches of reference lists. The search covered studies published between 2010 and 2026. Relevant studies published in English were identified using combinations of keywords such as "community-based interventions", "preventive health", "health promotion", "screening", "community health workers", and "population health", combined using Boolean operators (AND/OR) and adapted across databases. The identification and selection of studies followed a broad, iterative approach consistent with narrative reviews, prioritizing relevance to the research objective, diversity of contexts, and conceptual contribution rather than exhaustive systematic inclusion. Studies were included if they addressed community-based preventive strategies, reported health-related outcomes, and were conducted across diverse population settings, including low- and middle-income and high-income contexts. Exclusion criteria included purely clinical or hospital-based interventions without a community component and studies lacking outcome-related data. Study selection involved screening titles, abstracts, and full texts based on predefined eligibility criteria. Findings were synthesized thematically based on intervention type, implementation strategies, and reported outcomes. Although a formal meta-analysis was not undertaken, the heterogeneity of study designs and outcomes limited quantitative synthesis, and the quality and contextual relevance of included studies were considered during interpretation. Consistent with the narrative design, formal systematic review procedures such as protocol registration, exhaustive search validation, and comprehensive bias assessment were not applied. A formal risk of bias assessment was not conducted due to the narrative design. This approach ensured a structured yet flexible synthesis of evidence.

## Review

Conceptual frameworks underpinning community-based preventive interventions

Conceptual frameworks provide a theoretical structure for the design, implementation, and evaluation of preventive health strategies in local settings by clarifying the pathways through which change can be initiated and sustained [11,12]. The socio-ecological model has been foundational in understanding the formation of individual behaviors through interactions across interpersonal relationships, community contexts, institutional systems, and broader policy environments [19]. This interdisciplinary perspective enables integration of behavioral, environmental, and structural elements, ensuring that preventive approaches extend beyond individual-level education to address multilevel determinants of health [20]. Within this context, the framework is organized as a theory-integrated model that structures strategies across interconnected domains, including individual behavioral determinants, interpersonal and social processes, organizational and health system factors, and broader policy and environmental influences. These domains function as dynamically interacting components within a complex adaptive system rather than as isolated levels, enabling a more comprehensive understanding of intervention pathways and outcomes.

Frameworks from implementation science further strengthen conceptual clarity by offering structured approaches to evaluate reach, effectiveness, adoption, implementation fidelity, and sustainability of interventions [21]. The RE-AIM framework has been widely applied to assess real-world applications and guide scalability across diverse populations, with emphasis on both internal and external validity and long-term maintenance [13]. Integration of these constructs within the broader multilevel model enables alignment between theoretical domains and practical delivery components, facilitating cross-context comparison and identification of mechanisms influencing effectiveness and sustainability. Behavior change theories, including social cognitive and self-determination approaches, contribute to the design of lifestyle-focused strategies by addressing constructs such as self-efficacy, social support, and environmental reinforcement, which mediate the adoption of healthier behaviors [22]. The integration of behavioral theory with participatory approaches enhances cultural responsiveness and contextual adaptation [23].

Systems-oriented models further recognize that prevention operates within complex adaptive systems characterized by feedback loops, intersectoral interactions, and dynamic resource flows [21]. This perspective highlights the importance of coordination among health systems, governance structures, and civil society actors in sustaining outcomes. While conceptual and implementation models provide strong theoretical coherence and are widely applied across intervention types, the empirical strength of evidence linking specific frameworks to measurable population-level outcomes remains variable, with stronger support observed in integrated, multicomponent approaches compared to single-theory applications. The application of such integrated frameworks strengthens program design, supports evaluation, and facilitates translation of evidence into scalable and context-sensitive preventive strategies.

Community-based preventive strategies classification

Preventive strategies may be categorized based on prevention level, target population, delivery mode, and position within the broader health system [12]. Primary prevention aims to reduce exposure to risk factors before disease onset through health education, lifestyle modification, and environmental improvements implemented in accessible settings [24]. These approaches primarily address behavioral determinants linked to non-communicable diseases, including physical inactivity, unhealthy diet, tobacco use, and alcohol consumption [25].

Secondary prevention focuses on early detection and timely management through screening, risk assessment, and referral systems designed to identify undiagnosed conditions such as hypertension and diabetes at an early stage [15]. These strategies often rely on decentralized delivery models, including outreach campaigns and mobile health units, to improve access among underserved populations. Tertiary elements may also be incorporated, focusing on disease management, complication prevention, and recurrence reduction through peer support and adherence counseling mechanisms [26].

Preventive approaches may also be classified as individual-level, population-level, or policy-level based on their primary mechanism of action [11,21]. Individual-level strategies typically involve behavior change, communication, and skill development, whereas population-level approaches focus on social mobilization, capacity building, and participatory engagement processes [27]. Policy and structural measures include fiscal regulations and environmental modifications designed to influence contextual determinants of health behaviors. Across these categories, evidence is stronger for integrated, multilevel strategies that combine behavioral, screening, and policy components, whereas single-level approaches, particularly those limited to individual behavior change, demonstrate more variable and context-dependent effectiveness [28]. The key types of preventive measures are summarized in Table 1.

**Table 1:** Classification of community-based preventive strategies

Classification category	Description	Key characteristics	Reference
Primary prevention	Interventions aimed at preventing disease onset	Health education, lifestyle promotion, risk factor reduction	[1,20]
Secondary prevention	Early detection and timely management of asymptomatic conditions	Community screening, risk assessment, referral systems	[4,15]
Tertiary prevention	Prevention of complications and disease progression	Adherence counselling, peer support, chronic disease monitoring	[4,14]
Individual-level strategies	Target behavior change at the personal level	Counseling, skill-building, and self-management education	[2,17]
Policy/structural strategies	Modify environmental or regulatory contexts	Fiscal policies, environmental restructuring, and regulatory actions	[6,21]

Community engagement and participatory approaches

Community engagement is a central component of preventive health strategies in local settings, ensuring active involvement of populations in the planning, implementation, and evaluation of programs [27]. Participatory approaches position populations as active partners by incorporating contextual knowledge, cultural understanding, and social networks, thereby enhancing relevance, acceptability, and legitimacy [29]. Such involvement fosters trust, transparency, and shared ownership, which are essential for sustained population-level impact. Common participatory mechanisms include advisory boards, peer educators, and local leadership structures that align strategies with context-specific priorities and sociocultural norms [30]. These approaches also facilitate the identification of perceived health risks and barriers, enabling the design of context-specific responses. Evidence indicates that participatory models are associated with improved recruitment, adherence, and retention compared to externally designed programs. Additionally, they promote equity, collaborative decision-making, and capacity-building, contributing to empowerment through enhanced health literacy, leadership, and collective efficacy [31]. Effective implementation requires structured communication systems, transparent governance, and adequate resource allocation to ensure meaningful engagement.

**Figure 1:**
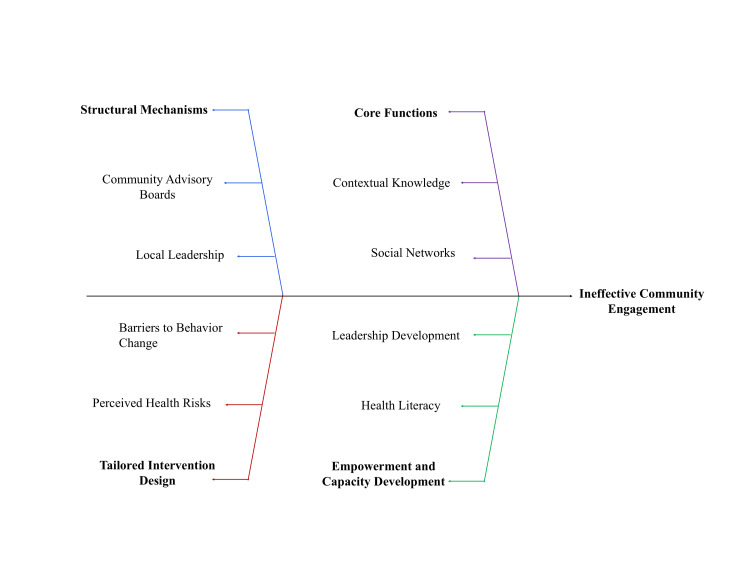
Determinants influencing community engagement effectiveness The figure illustrates the interaction between structural mechanisms, core functions, and empowerment processes, alongside behavioral barriers, in shaping the effectiveness of community engagement, with imbalances contributing to suboptimal outcomes.

These strategies may be further categorized into vertical, disease-specific programs and integrated, multisectoral approaches that address multiple risk factors simultaneously [21]. Integrated models are generally more effective at the population level due to their ability to address interconnected determinants of health. Comparatively, evidence for participatory approaches is strongest when embedded within integrated, multicomponent strategies that combine behavioral, service delivery, and structural interventions [32]. In contrast, standalone engagement efforts without linkage to service delivery or structural support show more limited and context-dependent impact on measurable outcomes. Such categorization supports clearer evaluation and facilitates cross-context comparison while aligning with frameworks guiding implementation and scale-up [33]. Overall, participatory approaches contribute substantially to acceptability and equity, although their direct effect on clinical outcomes remains less consistently demonstrated compared to screening or behavioral strategies. Figure 1 illustrates key functional, contextual, structural, and behavioral determinants influencing engagement effectiveness.

Role of community health workers and lay health facilitators

Community health workers (CHWs) and lay health facilitators are vital elements of preventive health systems, especially where workforce shortages and limited access to formal healthcare services persist [34]. These cadres serve as social bridges by addressing sociocultural and structural divides between health institutions and underserved populations through the provision of health education, risk assessment, screening, and referral services. Their proximity to populations enhances credibility, communication, and effectiveness in ensuring adherence to preventive recommendations [35]. Evidence indicates that CHW-led strategies contribute to improved hypertension and diabetes control, as well as management of other chronic conditions through follow-up, counseling, and linkage to care [36]. They also contribute to increased service coverage and early detection through involvement in immunization, maternal and child health, and infectious disease prevention programs. Lay facilitators further support behavioral adoption through culturally appropriate communication that accounts for language, literacy, and local beliefs [37].

Task-sharing models that incorporate CHWs enhance the capacity of primary health systems and improve access among marginalized groups. Structured training and supervision are essential to ensure quality, fidelity of implementation, and integration with formal health services [30]. Sustainable implementation requires stable financing, policy recognition, and standardized competency frameworks to ensure role clarity and accountability [21]. Integration of these workers within multidisciplinary teams supports continuity of care and enables population-level surveillance and data collection for monitoring preventive outcomes. In addition to service delivery, they contribute to advocacy and social mobilization, reinforcing broader preventive strategies [3]. Compared to other delivery mechanisms, CHW-led approaches demonstrate relatively strong evidence in improving service access, early detection, and treatment adherence, particularly in underserved settings, although effectiveness remains dependent on training quality, supervision, and system integration.

Behavior and lifestyle change interventions

Interventions targeting behavioral and lifestyle modification represent a core component of preventive strategies in local settings, addressing modifiable risk factors that contribute substantially to the global burden of disease [38]. These approaches target physical inactivity, unhealthy diets, tobacco use, harmful alcohol consumption, and sedentary behavior through structured education, training programs, and social support networks delivered in accessible environments. Such initiatives enhance participation and reduce structural barriers by situating activities within accessible contexts [2].

Lifestyle-focused strategies addressing obesity often draw on behavior change theories to promote self-efficacy, goal setting, peer support, and sustained motivation. Group-based activities, peer-led sessions, and culturally tailored communication have demonstrated positive effects on weight management, blood pressure, and glycemic control among high-risk populations [19]. Integrated programs combining nutrition education, physical activity promotion, and environmental modifications tend to produce more sustained outcomes compared to single-component approaches. The use of digital tools, including mobile applications and remote monitoring systems, has expanded reach and flexibility, enabling continuous engagement, personalized feedback, and behavioral tracking [17]. Collaboration with organizations further strengthens contextual adaptation and program ownership.

**Figure 2:**
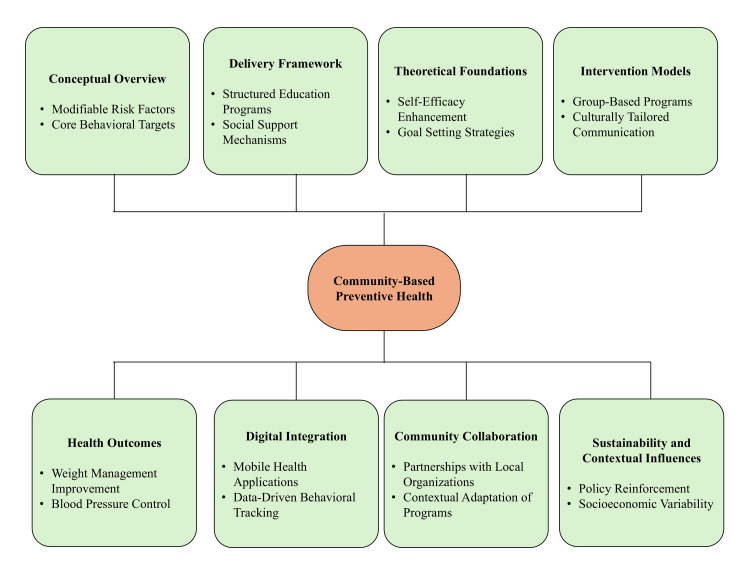
Components of community-based preventive health interventions

Sustained behavior change requires reinforcement through supportive policy and environmental conditions that enable healthier choices and reduce exposure to risk-promoting environments [16]. Variations in socioeconomic conditions, cultural practices, and resource availability influence effectiveness across settings. Evaluation of behavioral outcomes, adherence patterns, and long-term maintenance is necessary to determine population-level impact [20]. Compared to screening and policy-level approaches, lifestyle-focused strategies show strong evidence for short- to medium-term improvements in individual risk factors, whereas long-term sustainability remains more variable and dependent on continued engagement and supportive structural conditions. Figure 2 illustrates the conceptual, delivery, collaborative, digital, and contextual domains influencing outcomes.

Screening, early detection, and risk reduction programs

Screening, early detection, and risk reduction strategies in local settings are critical elements of preventive health programs, aimed at identifying asymptomatic disease and limiting progression before complications develop [4,15]. These activities are typically delivered through decentralized models that bring diagnostic and risk assessment services closer to populations with limited access to facility-based care [33]. Common initiatives focus on high-burden conditions such as hypertension, diabetes, cervical and breast cancer, and cardiovascular risk factors, which contribute significantly to premature morbidity and mortality [13].

Introducing screening services into accessible environments improves accessibility, reduces economic and geographical barriers, and facilitates earlier engagement with preventive care pathways [2]. Outreach approaches, including mobile clinics, door-to-door assessments, and health camps, are often used to reach underserved populations. Evidence indicates that structured screening initiatives improve case detection and enable timely linkage to diagnostic confirmation and treatment services [36]. Risk reduction components are frequently integrated, including counseling, lifestyle education, initiation of pharmacological treatment when required, and follow-up monitoring [36,38]. CHWs and lay facilitators play a central role in maintaining continuity of care through navigation support, appointment reminders, and adherence counseling [34]. Integration with primary healthcare systems strengthens referral pathways and aligns outreach activities with formal clinical management [10].

Data generated from these programs contribute to epidemiological surveillance and inform resource allocation at municipal and regional levels [11]. Increasing use of digital platforms supports documentation of screening outcomes, follow-up referrals, and longitudinal risk profiles, enhancing program accountability and efficiency. Implementation science frameworks assist in evaluating reach, effectiveness, and sustainability across diverse contexts [12]. Despite demonstrated benefits, challenges remain in maintaining quality assurance, diagnostic accuracy, and sustainable financing for large-scale implementation [24]. Variability in participation rates, follow-up adherence, and system integration continues to influence long-term impact [15,36]. Risk reduction efforts must be aligned with broader policy support, engagement mechanisms, and culturally responsive communication strategies to sustain participation and behavioral change [39].

Compared to behavior-focused approaches, screening and early detection strategies demonstrate stronger and more consistent evidence in improving early diagnosis and timely linkage to care, particularly for high-burden chronic conditions [36]. However, their effect on long-term morbidity and mortality remains more variable and is highly dependent on follow-up adherence, continuity of care, and health system integration. Evidence is strongest for integrated models that combine screening with risk reduction and follow-up mechanisms, whereas standalone initiatives without adequate referral and support structures show more limited effectiveness. These considerations are important in evaluating the role of screening and early detection in achieving equitable population health outcomes. Table 2 summarizes the key components of these strategies.

**Table 2:** Key components of screening, early detection, and risk reduction programs

Program component	Purpose	Implementation approach	Reference
Community-based screening	Identify undiagnosed conditions	Mobile clinics, outreach camps, and door-to-door assessment	[15,34]
Risk assessment tools	Detect high-risk individuals	Blood pressure checks, glucose testing, risk scoring	[4,14]
Referral linkage systems	Ensure continuity of care	Navigation support, follow-up coordination	[33]
Counselling and risk reduction	Promote behavioral modification	Lifestyle education, pharmacological initiation when required	[38]
Digital monitoring systems	Track outcomes and adherence	Electronic records, mobile-based follow-up platforms	[17,35]

Policy, environmental, and structural interventions at the community level

Policy, environmental, and structural approaches at the local level address upstream determinants of health by modifying the contexts in which individuals make behavioral decisions [40,41]. In contrast to individual-based programs, these strategies aim to transform regulatory frameworks, built environments, and economic incentives that shape exposure to risk factors across populations [16,42]. These approaches are grounded in population health principles, recognizing that individual responsibility is constrained by broader structural conditions [20].

Fiscal measures such as taxation of unhealthy commodities and subsidies for healthy foods have demonstrated measurable effects on consumption patterns and related health outcomes at both municipal and regional levels [40]. Regulatory actions, including restrictions on tobacco and alcohol availability and mandatory nutritional labeling, contribute to reducing exposure to behavioral risk factors across demographic groups [9]. Urban planning interventions that improve walkability, green spaces, and recreational infrastructure promote physical activity and reduce sedentary behavior [16]. Structural measures also extend to housing, sanitation, and transportation systems, which indirectly influence both communicable and non-communicable disease outcomes [4]. These actions typically require coordinated, multisectoral efforts involving health authorities, local governance, and planning agencies [6]. Implementation science frameworks support evaluation of adoption, fidelity, and sustainability across varying governance contexts [11,12].

Evidence indicates that environmental and structural modifications produce broader and more sustained population-level effects compared to isolated educational strategies, particularly when combined with engagement and service-based approaches [25,41]. Policy measures implemented alongside health promotion and screening initiatives demonstrate synergistic effects by reinforcing behavioral change within supportive environments [13,14]. However, implementation challenges include political resistance, economic trade-offs, and inequitable resource distribution [18,24]. Continuous monitoring is required to ensure that such measures do not unintentionally exacerbate disparities. Alignment of local policies with national health priorities supports sustained impact and long-term disease reduction. Compared to individual-level and screening-based approaches, system-level strategies demonstrate broader reach and more sustained influence on risk exposure, although the supporting evidence is often derived from observational and quasi-experimental studies, which may limit causal inference. Evidence is most robust when these measures are integrated with behavioral and service-based strategies, whereas isolated policy actions show more variable effectiveness depending on enforcement, governance context, and population compliance.

Influence on morbidity, mortality, and health behaviors

Preventive strategies in local settings are designed to produce measurable changes in morbidity, mortality, and behavioral risk patterns at the population level [1,42]. Multicomponent initiatives targeting cardiovascular risk factors have been associated with reductions in blood pressure, improved lipid profiles, and better glycemic control, contributing to decreased incidence of major adverse health events [14,36]. These findings indicate the capacity of coordinated preventive approaches to influence clinical indicators associated with premature mortality [4]. Behavioral pathways represent a primary mechanism through which these effects are realized, with structured initiatives promoting physical activity, dietary modification, smoking cessation, and reduced alcohol consumption demonstrating significant improvements in adherence to recommended practices [25]. Social support, self-efficacy, and collective norms within these contexts further reinforce sustained behavioral change [23].

Screening and early detection efforts contribute by reducing disease severity at diagnosis and enabling earlier initiation of treatment, which is associated with improved survival outcomes [14,15]. Continuity of care is strengthened through follow-up support provided by CHWs and participatory mechanisms, improving downstream clinical outcomes [8,34]. Integrated approaches combining behavioral education, screening, and environmental modification produce more cumulative effects than isolated strategies [21]. Improvements in risk factor control, healthcare utilization, and treatment adherence act as intermediate pathways influencing population-level mortality trends [1]. Longitudinal evidence suggests that sustained exposure to preventive measures can reduce disease burden over time, although variability in implementation fidelity, contextual adaptation, and resource allocation affects the magnitude of impact [12,18].

**Figure 3:**
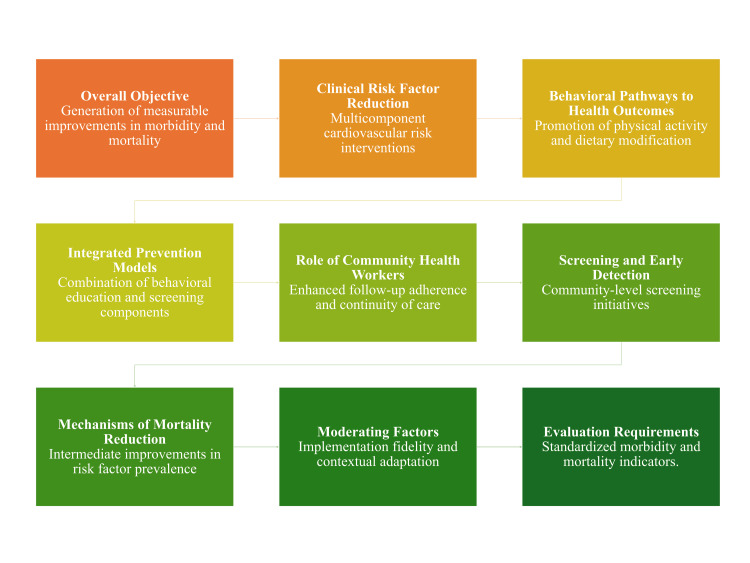
Framework of community-based prevention and health outcomes

Overall, the strongest evidence is observed for integrated, multicomponent approaches that combine behavioral, screening, and structural elements, demonstrating more consistent improvements in morbidity and risk factor profiles [14,21]. In contrast, single-component strategies show greater variability and context dependence, particularly in resource-limited or weak health system settings. Effective evaluation of outcomes requires standardized indicators, adequate follow-up duration, and integration of data systems with formal health information systems [11]. Continuous monitoring of behavioral trends and clinical endpoints is necessary to establish long-term effectiveness in improving population health outcomes. Figure 3 illustrates the relationships between preventive approaches, risk reduction, and health outcomes.

Equity, access, and social determinants of health

Equity represents a central consideration in preventive health strategies in local settings, as disparities in morbidity and mortality are closely linked to unequal distribution of social determinants such as income, education, housing, employment, and neighborhood conditions [43]. These approaches aim to reduce structural barriers that limit access to health-promoting resources and essential services among marginalized and underserved populations. Delivery within these contexts enables alignment with real-world conditions where health behaviors are shaped, and service utilization patterns emerge [44]. Factors such as geographic accessibility, financial constraints, cultural acceptability, language differences, and health literacy influence access to preventive services [45]. Outreach initiatives and involvement of CHWs strengthen delivery models by expanding coverage to populations often excluded from formal healthcare systems. Equity is further supported through participatory planning processes that incorporate local perspectives into program design and governance, enhancing responsiveness to identified priorities [46]. Efforts to address social determinants increasingly involve integrated approaches linking health services with housing support, food security programs, employment services, and transportation systems. Collaboration between social service providers, local governance structures, and public health agencies enhances the capacity to address overlapping determinants influencing health outcomes [47]. Evidence indicates that equity-focused initiatives can reduce disparities in screening uptake, chronic disease management, and preventive service coverage when supported by adequate resources and sustained implementation [48].

Effective monitoring requires disaggregated data to assess differential impacts across socioeconomic, gender, ethnic, and geographic groups. Implementation science frameworks can be applied to evaluate whether these strategies reduce or exacerbate existing disparities [49]. Persistent socioeconomic inequalities may limit the extent of improvement achievable without supportive policy reforms. Compared to domains such as screening and behavioral change, evidence related to equity outcomes is more heterogeneous and often indirect, with many studies demonstrating improvements in access and service utilization rather than consistent reductions in morbidity and mortality disparities [50]. Integrated models that combine behavioral, environmental, and structural components within an explicit equity framework show greater potential to reduce disparities and improve population health indicators. Evidence is strongest for multicomponent and intersectoral strategies addressing underlying social determinants, whereas isolated efforts targeting single access barriers tend to produce more limited and context-specific effects. Table 3 summarizes key determinants and corresponding response strategies.

**Table 3:** Equity, access, and social determinants in community-based prevention

Determinant/equity focus	Influence on health outcomes	Community-level response	Reference
Socioeconomic status	Differential exposure to health risks	Targeted outreach and subsidized services	[2,45]
Geographic accessibility	Limited proximity to healthcare services	Decentralized delivery and community outreach	[44]
Cultural and linguistic barriers	Reduced service utilization	Culturally tailored communication strategies	[29,37]
Health literacy	Limited understanding of preventive care	Community education and peer facilitation	[17]
Structural inequality	Persistent health disparities	Multisectoral collaboration and policy alignment	[6,50]

Comparative analysis of intervention effectiveness

Integrated analysis across intervention types indicates clear variation in the strength, consistency, and pathways of impact on population health outcomes. Multicomponent strategies that combine behavioral modification, screening, and structural or policy elements demonstrate the most robust and consistent evidence, reflecting their ability to address multiple determinants simultaneously and reinforce effects across levels. Screening and early detection programs show strong evidence for improving case identification, risk stratification, and timely linkage to care, although their long-term impact on morbidity and mortality remains dependent on follow-up adherence and health system integration. Behavioral and lifestyle interventions provide moderate evidence, particularly for short- to medium-term improvements in risk factors such as physical activity, diet, and metabolic indicators, but their sustainability is more variable in the absence of supportive environmental and policy conditions. CHW-led and participatory approaches contribute substantially to improving access, service utilization, and treatment adherence, especially in underserved populations, although their direct effects on clinical outcomes are more context-dependent and often mediated through integrated intervention components. Policy and structural interventions demonstrate broad population-level effects by modifying environmental exposures and behavioral contexts, with evidence suggesting sustained impact, although causal attribution is often derived from observational or quasi-experimental designs and influenced by governance and implementation capacity. Overall, evidence is strongest for integrated and system-oriented approaches, while single-component strategies, particularly those limited to individual behavior change or standalone engagement, show more variable and context-dependent effectiveness depending on implementation conditions and resource availability.

Limitations and future directions

There are a few methodological limitations inherent to the narrative design of this review, as it was not based on a formal systematic search strategy or meta-analytic synthesis, given the objective of providing a broad, integrative overview of diverse intervention types, contexts, and implementation approaches that are often heterogeneous and not directly comparable for quantitative synthesis. This review is not fully systematic in nature, and the processes of study identification and selection may therefore be subject to selection bias. Additionally, the potential influence of publication bias should be considered, as the available evidence may disproportionately reflect studies with favorable or statistically significant outcomes. As a result, the findings should be interpreted with caution, particularly in terms of completeness of evidence coverage and generalizability across settings. Inconsistencies in study design, intervention components, outcome measures, and evaluation models constrained cross-program comparison. Variation in implementation contexts, resource availability, and health system capacity also limits external validity. The lack of longitudinal data in a number of studies limited the ability to evaluate long-term population effects and cost-effectiveness.

Future studies should focus on standardized indicators of behavioral, clinical, and equity outcomes to improve cross-study comparability. Longitudinal and implementation-focused designs are required to assess sustainability and scalability across different contexts. Greater incorporation of economic evaluation and policy analysis would strengthen evidence-based decision-making. Methods incorporating mixed approaches, combining quantitative impact assessment with contextual process evaluation, can provide deeper insight into factors influencing effectiveness and equity in preventive interventions. These priorities reflect the multidimensional nature of prevention, spanning behavioral, health system, and policy domains, and necessitate comprehensive and integrated approaches in future investigations.

## Conclusions

This narrative review suggests that preventive health strategies in local settings represent an important approach to enhancing population health through coordinated, multilevel interventions. The integration of health promotion, lifestyle modification, screening, early diagnosis, and supportive policy measures has been associated with improvements in risk factor control and management of chronic diseases. Evidence is strongest for integrated, multicomponent interventions that combine behavioral, screening, and structural elements, while screening and early detection strategies demonstrate consistent effectiveness for improving case identification and timely linkage to care but show more variable impact on long-term morbidity and mortality. Behavioral and lifestyle interventions provide moderate evidence, particularly for short- to medium-term risk factor improvement, though sustainability remains less consistent without supportive environmental conditions. CHW-led and participatory approaches show strong evidence for improving access, service utilization, and adherence, but more limited and context-dependent effects on direct clinical outcomes. Policy and structural interventions demonstrate substantial population-level impact, particularly in modifying risk exposure, although supporting evidence is often observational and influenced by governance and implementation context. Engagement of CHWs and participatory approaches may enhance accessibility, cultural relevance, and sustainability of preventive services, particularly among underserved populations. Behavioral changes are further supported by environmental and structural measures that create enabling social and physical conditions. However, the sustainability and magnitude of impact are influenced by governance capacity, financing mechanisms, workforce preparedness, and alignment with broader health systems. Multisectoral collaboration and contextual adaptation remain critical to ensuring that these approaches address local determinants and reduce disparities. In contrast, evidence for single-component or isolated strategies remains more variable and context-dependent, with less consistent impact on long-term population health outcomes. These findings should be interpreted in light of the narrative design and heterogeneity of the included evidence. Strengthening implementation through standardized monitoring frameworks and long-term evaluation may improve effectiveness and scalability. Accordingly, conclusions should be interpreted with consideration of variability in study designs, implementation fidelity, and contextual differences across settings. Overall, such approaches offer a promising pathway for reducing disease burden and improving population health outcomes across diverse settings.
